# Quality improvement practices used by teaching versus non-teaching trauma centres: analysis of a multinational survey of adult trauma centres in the United States, Canada, Australia, and New Zealand

**DOI:** 10.1186/1471-2482-14-112

**Published:** 2014-12-22

**Authors:** Vikas P Chaubey, Derek J Roberts, Mauricio B Ferri, Niklas H Bobrovitz, Henry T Stelfox

**Affiliations:** Department of Critical Care Medicine, University of Calgary, Foothills Medical Centre, McCaig Tower, 3134 Hospital Drive NW, Calgary, AB T2N 2T9 Canada; Department of Surgery, University of Calgary, Foothills Medical Centre, North Tower, 10th Floor, 1403 - 29th Street NW, Calgary, AB T2N 2T9 Canada; Department of Community Health Sciences, University of Calgary, Teaching, Research & Wellness Building, 3rd Floor, 3280 Hospital Drive NW, Calgary, AB T2N 4Z6 Canada

**Keywords:** Trauma quality improvement, Teaching status, Survey

## Abstract

**Background:**

Although studies have suggested that a relationship exists between hospital teaching status and quality improvement activities, it is unknown whether this relationship exists for trauma centres.

**Methods:**

We surveyed 249 adult trauma centres in the United States, Canada, Australia, and New Zealand (76% response rate) regarding their quality improvement programs. Trauma centres were stratified into two groups (teaching [academic-based or –affiliated] versus non-teaching) and their quality improvement programs were compared.

**Results:**

All participating trauma centres reported using a trauma registry and measuring quality of care. Teaching centres were more likely than non-teaching centres to use indicators whose content evaluated treatment (18% vs. 14%, p < 0.001) as well as the Institute of Medicine aim of timeliness of care (23% vs. 20%, p < 0.001). Non-teaching centres were more likely to use indicators whose content evaluated triage and patient flow (15% vs. 18%, p < 0.001) as well as the Institute of Medicine aim of efficiency of care (25% vs. 30%, p < 0.001). While over 80% of teaching centres used time to laparotomy, pulmonary complications, in hospital mortality, and appropriate admission physician/service as quality indicators, only two of these (in hospital mortality and appropriate admission physician/service) were used by over half of non-teaching trauma centres. The majority of centres reported using morbidity and mortality conferences (96% vs. 97%, p = 0.61) and quality of care audits (94% vs. 88%, p = 0.08) while approximately half used report cards (51% vs. 43%, p = 0.22).

**Conclusions:**

Teaching and non-teaching centres reported being engaged in quality improvement and exhibited largely similar quality improvement activities. However, differences exist in the type and frequency of quality indicators utilized among teaching versus non-teaching trauma centres.

## Background

Quality improvement programs are an important component of trauma centre and system structure [1], and have been shown to be a valuable administrative tool to strengthen the care of severely injured patients [2]. However, at present, trauma centres appear to conduct quality improvement programs of varying degrees of intensity and sophistication [3]. Despite this heterogeneity, it remains largely unknown whether quality improvement program structure and activities improve overall trauma patient outcomes [4, 5].

Conflicting evidence exists regarding the effect of hospital teaching status on patient care. Although teaching hospitals have higher volumes, which may correlate with improved outcomes [6, 7], this association may be negated by the presence of learners and their relative inexperience. Moreover, while a systematic review reported that hospital teaching status had little effect on patient outcomes, this association may depend on the disease examined [8]. Only one study has examined the association between outcomes of injured patients and teaching status. This study of splenic injury management found that teaching hospitals were more likely to attempt non-operative management, which resulted in increased rates of splenic salvage [9].

Although quality improvement has been used in trauma care for some time, there exists a gap in knowledge whether teaching trauma centres differ in their quality improvement activities relative to non-teaching centres. A recent review suggested that teaching hospitals had superior quality indicator use in terms of process measurement and other non-mortality end points relative to those used by non-teaching centres, but did not focus specifically on trauma care [10]. We recently conducted a large multinational survey specifically designed to assess the quality indicators used by trauma centres [5]. This study reports the results of a re-analysis of this survey to explore the relationship between trauma quality improvement programs and hospital teaching status.

## Methods

To examine the quality of care provided to severely injured patients, we developed a conceptual model of quality indicators in trauma care that merges Donabedian’s framework of quality of care with modern systems of trauma care. This was previously described in a scoping review of quality indicators in trauma care [11]. A survey tool was developed based on the results of this scoping review and semi-structured interviews with injury and quality of care experts. The details of survey development, design, implementation, and data collection have previously been outlined [5, 12]. A copy of the survey is available online and can be found at: http://links.lww.com/TA/A93. Ethics approval was obtained from the Conjoint Health Research Ethics Board at the University of Calgary.

The original study included a voluntary, web-based cross-sectional survey of 330 trauma centre program leaders between March 10, 2009 and June 19, 2009 in the United States, Canada, Australia, and New Zealand. The survey collected information on trauma centre level of care designation, geographic location, teaching status, number and type of injured patients managed, nature of their quality improvement program, and quality indicators utilized [5]. The Internet was also searched for quality improvement data on surveyed trauma centre websites. Analyses were performed with trauma centres classified into two self-reported groups: teaching (university based teaching setting or university affiliated teaching setting) and non-teaching (non-teaching setting). Trauma centres were categorized as high volume according to American College of Surgeons (ACS) annual volume requirements for a Level I centre of at least 1,200 patients with any Injury Severity Score (ISS) [13], and at least 240 patients with ISS >15 [1].

### Statistical analysis

The strategy for the primary analysis was to describe and compare the quality improvement programs of trauma centres according to teaching status. Medians were used when distributions were skewed and contained several outliers. Comparisons of dichotomous responses and derivation of confidence intervals for teaching versus non-teaching trauma centres were performed using the Chi-squared test. In order to assess for effect measure modification/subgroup differences, we stratified these dichotomous outcomes by trauma centre accreditation/verification, ACS level of care designation, geographic location, volume, median household income of the surrounding neighborhoods, and the number of patients assessed yearly. The Mann–Whitney *U*-test was used for comparisons of data summarized using medians. Statistical analyses were conducted using Stata version 10 (Stata Corp, San Antonio, TX).

## Results

The survey was sent to 330 trauma centres (263 in the United States, 46 in Canada, 18 in Australia, and 3 in New Zealand) between March 10, 2009, and June 19, 2009, and 251 (76%) responded. Of the 251 centres that responded, 174 (69%) were teaching and 75 (30%) were non-teaching centres, and 2 (<1%) could not have their teaching status classified due to missing data. All the participating trauma centres reported using a trauma registry. The characteristics of the trauma centres responding to the survey are summarized in Table 1 stratified according to teaching status.

**Table 1:** **Characteristics of trauma centres participating in survey as stratified by teaching status** ^*^

Characteristic	Teaching (%)	Non-teaching (%)	Chi-square
	(N = 175)	(N = 74)	P-Value
Accredited or verified as:	(N = 155)^*^	(N = 70)^*^	
Trauma centre	140 (90)	65 (93)	0.37
Trauma system	4 (2.6)	3 (4.3)	
Trauma centre & system	11 (7.1)	2 (2.9)	
Level of designated care	(N = 171)^*^	(N = 68)^*^	
Level 1	152 (89)	40 (59)	<0.001
Level 2	15 (8.8)	18 (26)	
Level 3 or 4	4 (2.3)	10 (15)	
Geographic Location	(N = 175)^*^	(N = 74)^*^	
Urban	151 (86)	46 (62)	<0.001
Suburban	15 (8.6)	15 (20)	
Rural	9 (5.1)	13 (18)	
Volume	(N = 99)^*^	(N = 47)^*^	
Low	43 (43)	41 (87)	<0.001
High	56 (56)	6 (13)	
Median household income of surrounding neighbourhoods	(N = 175)^*^	(N = 74)^*^	
Top tertile	59 (34)	23 (31)	<0.001
Middle tertile	46 (26)	38 (51)	
Bottom tertile	70 (40)	13 (18)	
Designated trauma team	170 (97)	71 (96)	0.62
Trauma team activation criteria	168 (96)	72 (97)	0.62
Patients with injuries assessed yearly			
Any ISS, median (IQR)	1733 (930–2652)	890 (468–1412.5)	<0.001^**^
ISS > 15, median (IQR)	402 (227–638)	132 (73.5-225)	<0.001^**^
Trauma registry	173 (99)	71 (96)	0.13

IQR: interquartile range.

ISS: Injury Severity Score.

^*^Complete information was not available for all centres. The number of survey responses is presented if different from the total sample.

^**^The Mann–Whitney *U*-test was used for calculating this p-value.

### Performance measurement

The content of the quality indicators and the Institute of Medicine dimensions of care evaluated by the quality indicators are summarized in Table 2. With respect to the content of the quality indicators, teaching centres were more likely to use indicators for evaluating treatment (18% vs. 14%, p < 0.001) and non-teaching centres more likely to use indicators evaluating triage and patient flow (15% vs. 18%, p < 0.001). With respect to the Institutes of Medicine dimensions of care, teaching centres were more likely to use indicators evaluating timeliness of care (23% vs. 20%, p < 0.001) and non-teaching centres were more likely to use indicators evaluating efficiency of care (25% vs. 30%, p < 0.001).

**Table 2:** **Quality indicator use according to teaching status** ^**†**^

Quality indicators	Teaching (%)	Non-teaching (%)	Chi-square
			P-value
Trauma centres measuring quality indicators	173 (99)	72 (97)	0.37
No. quality indicators			
Total	5557	2934	
Median number per centre (IQR)	26 (14,38)	21.5 (10–43.5)	p = 0.556^*^
Donabedian dimensions of care evaluated by quality indicators			
Structure	45 (0.008)	27 (0.009)	0.96
Process	3696 (67)	2027 (69)	0.02
Outcome	1825 (33)	889 (30)	0.02
Content of the quality indicators^**^			
Medical error or adverse event (e.g. decubitus ulcer rate)	1829 (33)	913 (31)	0.09
Treatment (e.g. time to treatment of joint dislocation)	1013 (18)	413 (14)	<0.001
Triage & patient flow (e.g. field triage rate)	823 (15)	539 (18)	<0.001
Documentation (e.g. invasive prehospital procedure documentation rate)	561 (10)	357 (12)	0.003
Clinician(s) (e.g. trauma team activation for all major injuries)	335 (6.0)	190 (6.5)	0.42
Morbidity & mortality (e.g. hospital mortality rate)	255 (4.4)	139 (4.7)	0.76
Diagnostic studies & patient monitoring (e.g. time to CT scan)	269 (4.8)	122 (4.2)	0.15
Pre-hospital times (e.g. scene time)	192 (3.5)	107 (3.6)	0.65
Other (e.g. attendance at morbidity and mortality rounds)	134 (2.4)	90 (3.1)	0.07
IOM dimensions of care evaluated by quality indicators^**^			
Safe (e.g. rate of unplanned intensive care unit admissions)	2667 (48)	1441 (49)	0.33
Effective (e.g. rate of deep vein thrombosis prophylaxis)	1733 (31)	973 (33)	0.06
Patient-Centred (e.g. acute pain management for all injured patients)	33 (0.59)	16 (0.55)	0.78
Timely (e.g. time to acute subdural hematoma evacuation)	1302 (23)	577 (20)	<0.001
Efficient (e.g. field triage rate)	1395 (25)	879 (30)	<0.001
Equitable (e.g. deaths referred for potential organ donation)	1 (0.018)	0 (0)	0.47

CT: computed tomography.

IOM: Institute of Medicine.

IQR: interquartile range.

^†^Data reported as number (percentage) unless otherwise indicated.

*Calculated by the Mann Whitney *U* test.

**Missing data accounted for <1% of all data and complete case analyses were performed.

The top 10 quality indicators used by teaching centres compared to non-teaching centres are summarized in Table 3. Seven of the top 10 quality indicators were common to both teaching and non-teaching centres. All 10 indicators were used by over half of the teaching centres and more than 80% of teaching centres used the following four indicators: time to laparotomy, pulmonary complications, in hospital mortality, appropriate admission service/physician. Conversely, three indicators were used by over half of the non teaching centres with the most common, in hospital mortality used by 57% of non-teaching centres.

**Table 3:** **Top 10 quality indicators used by teaching vs. non-teaching centres** ^*^

	Teaching centres		Non-teaching centres	
Rank	Quality indicator	Frequency (%)	Quality indicator	Frequency (%)
1	Appropriate admission service/physician	96	In hospital mortality	57
2	In hospital mortality	93	Attendance at M & M conference	54
3	Pulmonary complications	86	Appropriate admission service/physician	50
4	Time to laparotomy	81	Scene time	44
5	Length of stay	72	Trauma team activation for severely injured patients	41
6	Scene time	69	Pulmonary complications	40
7	Time to craniotomy in severe TBI	68	Length of stay	36
8	Secure airway for comatose patient	56	Inter-facility patient transfer	34
9	Reintubation <48 hours of extubation	55	Time to laparotomy	32
10	Attendance at M & M conference	54	Under triage	32

M&M: morbidity and mortality.

TBI: traumatic brain injury.

^*^Shaded boxes represent quality indicators common to both teaching and non-teaching centres.

### Quality improvement

The quality improvement practices according to trauma centre teaching status are summarized in Table 4. Trauma centre quality improvement practices appeared to be similar across centres of different teaching status. The majority of teaching versus non-teaching centres reported using morbidity and mortality conferences (96% vs. 97%, p = 0.61), quality of care audits (94% vs. 88%, p = 0.08) and both internal (79% vs. 77%, p = 0.77) and external (76% vs. 69%, p = 0.22) benchmarking. Approximately half of teaching and non-teaching centres (51% vs. 43%, p = 0.22) reported using report cards. Teaching centres were more likely to participate in research.

**Table 4:** **Quality improvement practices according to teaching status** ^*^

Characteristic	Teaching (%)	Non-teaching (%)	Chi-square
	(N = 175)	(N = 74)	P-value
Quality improvement practices	(N = 174)^*^	(N = 74)^*^	
Morbidity & mortality conferences	167 (96)	72 (97)	0.61
Quality of care audits	164 (94)	65 (88)	0.08
Report cards	90 (51)	32 (43)	0.22
Internal benchmarking	137 (79)	57 (77)	
External benchmarking	133 (76)	51 (69)	0.22
Research	(N = 173)^*^	(N = 73)^*^	
Local investigator initiated	28 (16)	10 (14)	0.62
Multi-centre investigator initiated	66 (38)	9 (12)	<0.001
Industry sponsored	61 (35)	8 (11)	<0.001
Do not participate	18 (10)	46 (63)	<0.001

^*^Complete information was not available for all centres and the number of survey responses is presented for each characteristic.

### Subgroup analyses

The results were similar when stratified by trauma centre accreditation/verification, ACS level of care designation, geographic location, volume, median household income of the surrounding neighborhoods, and the number of patients assessed yearly.

## Discussion

Teaching and non-teaching centres reported being engaged in quality improvement and reported similar quality improvement activities. Small differences in the types of quality indicators used by centres were observed according to teaching status. Teaching centres were more likely to use indicators evaluating treatment and timeliness of care, while non-teaching centres were more likely to use indicators evaluating triage and patient flow as well as efficiency of care. Teaching centres were more likely to use the same indicators than non-teaching centres.

Present medical literature suggests that there does not appear to be any differences in patient outcomes in teaching versus non-teaching environments [8]. The literature is, however, limited by its observational nature, heterogeneity, and overall low quality [8]. With respect to quality improvement programs, there is little known about differences between teaching and non-teaching centres. One study has suggested that teaching centres have better quality of care measures than non-teaching centres in terms of processes of care and other non-mortality outcomes [10].

Interestingly, there were few large differences documented between teaching and non-teaching centres in our study despite potentially important differences in their characteristics (e.g., level of designation, geographical location, surrounding neighborhoods, number and nature of patients). It is conceivable that because the ACS mandates accredited trauma centres to partake in quality improvement activities, this leads to some homogeneity across institutions in the overall strategies. Previously published work describes in greater detail the quality indicators (QIs) that trauma centers use for quality measurement and performance improvement *[5, 14].* However, there appear to be small but potentially important differences in trauma centre performance measurement and quality improvement between teaching and non-teaching centres.

The results of our study paralleled those from a previous study analyzing trauma centre volume and quality improvement programs [12]. As would be expected, non-teaching centres were more likely to be low-volume centres located in suburban and rural settings with a higher proportion of middle-income neighbourhoods surrounding these hospitals. Teaching centres were more likely to be high-volume centres located in urban settings with a higher proportion of lower income neighbourhoods surrounding these hospitals.

Although teaching status segregated closely with volume status there were differences noted when stratifying by each of these categories. Teaching centres were more likely to use indicators for evaluating treatment and timeliness of care whereas high volume centres placed a greater emphasis on measurement of medical errors and adverse events, the use of guidelines and protocols, and employing report cards and benchmarking as quality improvement tools [12]. Non-teaching centres were more likely to use indicators to evaluate triage and patient flow and efficiency of care. Low volume centres measured the same quality indicators but in addition they were also more likely to measure effectiveness of care [12].

The top 10 quality indicators were more likely to be used by teaching centres relative to non-teaching centres (>80% of teaching centres used time to laparotomy, pulmonary complications, in hospital mortality, appropriate admission service/physician). The quality indicators used in teaching centres versus non-teaching centres may reflect patterns specific to the volume of patients they each encounter and the types of services available. Thus quality indicator use is targeted to local quality of care challenges. For instance, 68% of teaching centres measured time to craniotomy whereas this was not one of the top 10 quality indicators for non-teaching centres; perhaps an indication of higher volume centres having the availability of neurosurgical services. On the other hand, 41% of non-teaching centres measured trauma team activation for severely injured patients whereas this was not one of the top 10 quality indicators for teaching centres; perhaps a reflection of challenges faced in smaller volume centres with the consistency with trauma team activation.

This study has several limitations, including its reliance on volunteer survey participants whose quality improvement activities may differ from centres that did not participate in the survey, the simplicity of the survey (high level description of quality improvement activities), and the lack of patient outcome data relating to morbidity and mortality. Differences in performance measurement and quality improvement could be associated with patient outcomes and warrants further evaluation. Moreover, as we conducted multiple statistical tests, one or more of our observed associations could have been due to chance alone. Further studies should assess the relative importance of the different facets of quality improvement on patient outcomes and how they interact with institutional characteristics so that professional trauma organizations can accurately recommend the best quality improvement processes.

## Conclusions

Our study provides the first examination of trauma centre quality improvement programs according to trauma centre teaching status. Our data indicates that most trauma centres are engaged in quality improvement employing a diverse range of performance measures and improvement strategies. However, there appear to be small but potentially important differences in trauma centre performance measurement and quality improvement according to trauma teaching status.

## Abbreviations

ACS: American College of Surgeons
ISS: Injury severity score
CIHR: Canadian Institutes of Health Research
AIHS: Alberta Innovates Health Solution.
